# Minimization of free radical damage by metal catalysis of multivitamin/multimineral supplements

**DOI:** 10.1186/1475-2891-9-61

**Published:** 2010-11-23

**Authors:** Alexander B Rabovsky, Andrei M Komarov, Jeremy S Ivie, Garry R Buettner

**Affiliations:** 1 Research & Technology Development, Melaleuca Inc. Idaho Falls, ID, 83402, USA; 2 Department of Biochemistry and Molecular Biology, The George Washington University, Washington D.C. 20037, USA; 3 Free Radical and Radiation Biology, The University of Iowa, Iowa City, IA 52242, USA

## Abstract

Multivitamin/multimineral complexes are the most common dietary supplements. Unlike minerals in foods that are incorporated in bioorganic structures, minerals in dietary supplements are typically in an inorganic form. These minerals can catalyze the generation of free radicals, thereby oxidizing antioxidants during digestion. Here we examine the ability of a matrix consisting of an amino acid and non-digestible oligosaccharide (AAOS) to blunt metal-catalyzed oxidations. Monitoring of ascorbate radical generated by copper shows that ascorbate is oxidized more slowly with the AAOS matrix than with copper sulfate. Measurement of the rate of oxidation of ascorbic acid and Trolox^® ^by catalytic metals confirmed the ability of AAOS to slow these oxidations. Similar results were observed with iron-catalyzed formation of hydroxyl radicals. When compared to traditional forms of minerals used in supplements, we conclude that the oxidative loss of antioxidants in solution at physiological pH is much slower when AAOS is present.

## 1.0 Introduction

There is increasing interest by the public in nutrition, functional foods, and nutritional supplements. The nutritional supplement market in the United States is estimated to be over $1 × 10^10 ^y^-1 ^and growing [1]. Although dietary supplements are not intended to substitute for a healthy variety of food, millions of people complement their daily food intake with dietary supplements to ensure the requisite intake of essential nutrients required for proper bodily functions and good health. Formulations of multivitamin supplements typically include oxidation-sensitive vitamins, such as vitamin C and E, as well as minerals, such as iron and copper, in the same formulation. Minerals can have limited solubility, depending on their exact form. In addition redox active transition metals, such as iron and copper, can serve as catalysts for the oxidation of organic compounds. For example, adventitious, trace levels of iron and copper in near-neutral phosphate buffer readily catalyze the oxidation of ascorbate [2,3]. Ferric iron is a standard reagent used to oxidize tocopherols to their corresponding quinones [4]. Thus, these metals could bring about the loss of antioxidants before absorption by the digestive system.

The rate of metal-catalyzed oxidations, *e.g*. by copper or iron ions, varies greatly with solubility and the ligand environment. In addition, the metal-catalyzed oxidation of ascorbate can lead to the oxidation of other substances in the solutions [5]. In fact the combination of iron and ascorbate has long been used to oxidize organics; the combination of these two reagents is referred to as the Udenfriend system and is used to for the hydroxylation of alkanes, aromatics, and other oxidations [6,7]. The combination of iron and ascorbate has also been used as a tool to initiate oxidations in cells, especially the oxidation of cellular structures that have unsaturated lipids [8]. As might be predicted, the production of hydroxyl radical has been observed upon dissolution of supplement tablets containing ascorbate [9]. Co-supplementation of ferrous salts with vitamin C can increase oxidative stress in the gastrointestinal tract, reviewed in [10]. Thus, a challenge is to provide a multivitamin/multimineral formulation that facilitates solubilization of the minerals and at the same time blunts the propensity of redox active metals to catalyze unwanted oxidations.

Here we investigate the ability of the supporting matrix in supplemental minerals to minimize the metal-catalyzed oxidation of oxidation-sensitive vitamins, *e.g*. ascorbate and tocopherol. Fructose-based oligosaccharides, such as inulin, have been demonstrated to enhance the absorption of calcium, magnesium, iron, and zinc [11-13]. Intake of inulin is associated with positive health effects, including maintenance of bone structure [14] and bone mineral content [15,16]. Here we examine the ability of this oligosaccharide in combination with amino acids (AAOS) to blunt metal-catalyzed oxidation of antioxidants.

## 2.0 Materials and methods

### 2.1 Materials

5,5-Dimethylpyrroline-1-oxide (DMPO; CAS# 3317-61-1), 2',7'-dichlorodihydrofluorescein diacetate (CAS# 4091-99-0), and ascorbic acid (50-81-7) were from Sigma Chemical Co. (St. Louis, MO); Trolox^® ^(53188-07-1) was from Aldrich (Milwaukee, WI). Cupric carbonate(CAS# 12069-69-1), copper sulfate (CAS# 7758-99-8), copper glycinate (CAS# 13479-54-4), ferrous sulfate (CAS# 13463-43-9), glycine (CAS# 56-40-6), L-aspartic acid (CAS# 56-84-8), copper gluconate (CAS# 527093), and inulin (molecular weight of approximately 5,000 Da, CAS# 9005-80-5) were from Spectrum Chemicals & Laboratory Products, (New Jersey).

### 2.2 Amino Acid Oligosaccharide (AAOS) Matrices

**2.2.1 **The copper-AAOS system was prepared by suspending Cu carbonate with glycine or aspartic acid followed by inulin at final molar ratio 1:4:0.01. After stirring for 10 min at 80°C the mixture was dried in an oven. Absence of carbonate was confirmed with hydrochloric acid.

**2.2.2 **The iron-AAOS system was prepared by first dissolving FeSO_4 _(1 mol) in water; then NaOH was added to precipitate the iron. Glycine or aspartic acid (2 mol) was added to a suspension of the Fe-solids; the mixture was stirred and then dried in oven. To prepare the iron-AAOS matrix glycine or aspartic acid (1 mol) was suspended in water with the iron solids; then 0.01 mol of inulin was added. After heating at 80°C, the resulting mixture was dried in oven.

### 2.3 EPR Spectroscopy

All EPR measurements were done using a Bruker ER-200 X-band EPR spectrometer. Samples (50 μL) in capillary tubes (0.5 mm i.d.) were examined at room temperature. EPR instrument settings were: (1) for ascorbate experiments - microwave frequency 9.71 GHz; center field 3472 G; scan rate 10 G/20 s; modulation amplitude 1.25 G; time constant 0.5 s; microwave power 10 mW; and instrument gain 2 × 10^6^; (2) for DMPO spin trapping (hydrogen peroxide plus iron or copper) - microwave frequency 9.71 GHz; center field 3472 G; scan rate 100 G/100 s; modulation amplitude 1.25 G; time constant 0.5 s; microwave power 10 mW; instrument gain was 2 × 10^6^; (3) for Trolox^® ^experiments - microwave frequency 9.71 GHz; center field 3472 G; scan rate 60 G/50 s; mod amp 1.0 G; time constant 0.5 s; microwave power 20 mW; and instrument gain 2 × 10^6^; and (4) for transition metals - microwave frequency 9.71 GHz; center field 3415 G; scan rate 1000 G/100 s; modulation amplitude 2.5 G; time constant 0.5 s; microwave power 10^2 ^mW; instrument gain varied for different samples from 1.0 × 10^3 ^to 3.2 × 10^5^. Manganese in calcium oxide was used as a reference standard.

### 2.2 Dichlorodihydrofluorescein Oxidation

2',7'-Dichlorodihydrofluorescein diacetate was hydrolyzed in 20 mM NaOH at room temperature for 20 min to remove the acetate esters to produce 2',7'-dichlorodihydrofluorescein (DCFH_2_). Mineral stock solutions were prepared in de-ionized water. DCFH_2 _(200 μL of 90 μM in 20 mM carbonate buffer, pH 7.0) and 80 μL of mineral solution (100 μM in 20 mM carbonate, pH 7.0) were mixed in a standard UV-Vis cuvette. The reaction was initiated by addition of 20 μL of 0.3% H_2_O_2 _yielding a final concentration of 88 mM. Absorbance at 500 nm (ε_500 _= 59,500 M^-1 ^cm^-1 ^[17] was monitored for 30 min.

## 3.0 Results and Discussion

### 3.1 EPR of Copper Complexes

**Figure 1:**
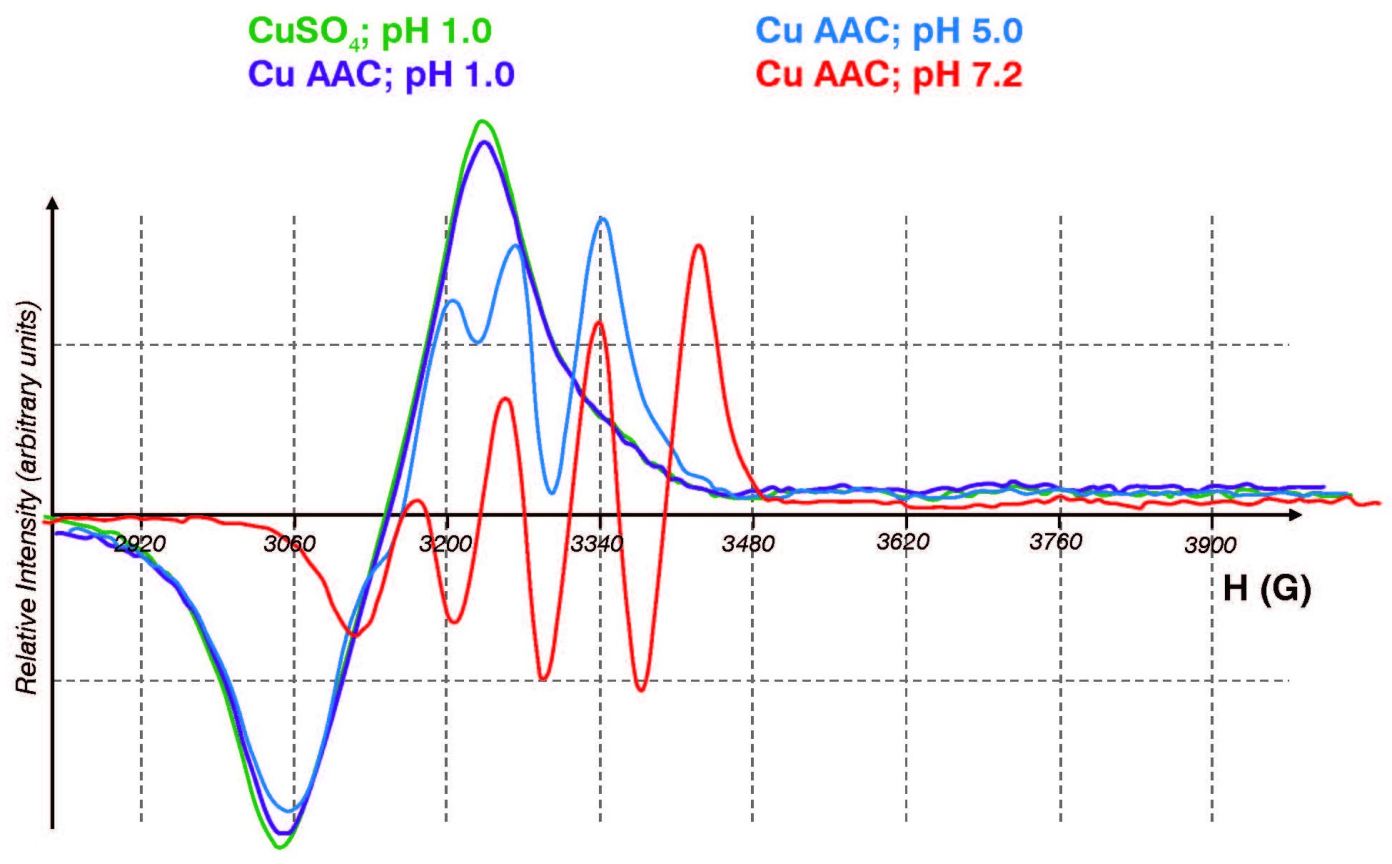
**EPR spectra of copper(II) compounds in different pH environments**. The copper (Cu^2+^) solutions were 5 mM in de-ionized water. The pH was adjusted with HCl or NaOH as appropriate. At pH 1, both forms of copper yield a single-line spectrum, g = 2.19, ΔH_pp _≈ 140 G. At pH 7, Cu(II)AAC has a g-value of 2.12 with a copper hyperfine splitting of 63 G.

To probe the nature of the aqueous copper complexes at different pH values we used EPR spectroscopy. When either CuSO_4 _or CuAAC (5 mM) was dissolved in aqueous solution at pH 1, the resulting EPR spectra were single lines (g = 2.19; ΔH_pp _= 140 G), **Figure **1. Similar spectra were observed when these complexes were dissolved in 0.5 M perchloric acid (not shown) consistent with the aqua-complex of copper. However, at near-neutral pH, the EPR of the ACC complex changed to that expected for a histidine-type complex [18]. This indicates that at neutral pH, typical amino acids can become ligands to copper(II).

### 3.2 Ascorbate Oxidation is Slowed by AAOS

**Figure 2:**
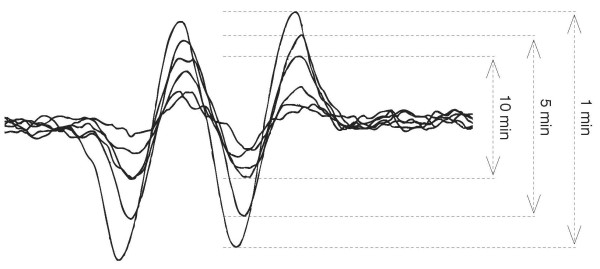
**The EPR spectrum of the ascorbate free radical at different times**. g-factor (g) - 2.005; Hyperfine splitting constant (a^H^) - 1.8 G

**Figure 3:**
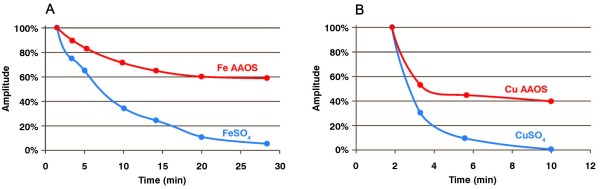
**AAOS slows both iron- and copper-catalyzed oxidation of ascorbate, compared to their sulfate forms**. Loss of ascorbate free radical vs. time in different environments. Aqueous solutions (20 mM HEPES pH 7.2) contained 2.0 mM ascorbic acid. **Panel A **- FeSO_4 _(0.45 mM); **Panel B **- CuSO_4 _(0.39 mM).

Ascorbate readily oxidizes in aerated aqueous solutions. The rate is a function of pH, the higher the pH the greater the rate of autoxidation. However, in acidic and near-neutral solutions, the rate of ascorbate oxidation is principally controlled by the concentration of catalytic transition metals [2,19,20]. The rate of ascorbate oxidation is reflected in the rate of oxygen consumption as well as the concentration of the one-electron oxidation product, the ascorbate free radical [21]. To determine if AAOS slows the rate of iron- and copper-catalyzed oxidation of ascorbate we used EPR to determine the level and time course of ascorbate radical formation in near-neutral solutions of ascorbate, **Figure **2. As anticipated, the introduction of iron or copper to near-neutral solutions of ascorbate resulted in an increase of the concentration of the ascorbate radical **Figure **3. This is consistent with these metals catalyzing the oxidation of ascorbate. As the concentration of ascorbate decreases, the concentration of the ascorbate radical will decrease [21,22]. We observed that the rate of loss of the ascorbate radical was significantly slowed by AAOS compared to sulfate forms of these metals. These results indicate that the AAOS complex slows the catalytic oxidation of ascorbate by copper and iron.

### 3.3. AAOS Blunts Formation of DMPO/^‧^OH

**Figure 4:**
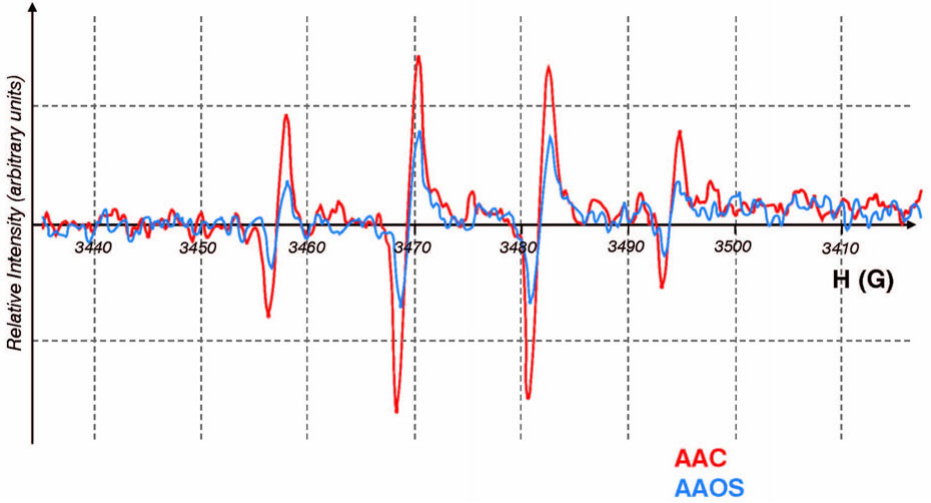
**AAOS blunts formation of DMPO/^‧^OH**. Hydroxyl radical was generated by the Fenton reaction in aqueous solutions (20 mM HEPES pH 7.2) with DMPO (20 mM), H_2_O_2 _(60 mM), Fe (0.9 mM), or copper (0.8 mM).

The experiments monitoring the ascorbate radical suggest that AAOS will suppress the oxidative chemistry of redox active metals. To further test this we used EPR spin trapping with 5,5-dimethylpyrroline-1-oxide. Reduced metals, such as Fe^2+^, will react with hydrogen peroxide; this reductive reaction (Fenton reaction) will generate the hydroxyl free radical [23], which will react with DMPO yielding a unique EPR-detectable spin adduct [24]. Indeed when this reaction was initiated with Fe-ACC a robust EPR signal consistent with the formation of DMPO/**^‧^**OH was observed, **Figure **4. However, when this same reaction was initiated with iron in an AAOS matrix, the EPR signal of DMPO/**^‧^**OH was reduced by over 60%. Parallel experiments with copper, showed similar results (a reduction of 50%), not shown. Thus, the AAOS matrix blunts the formation of DMPO/**^‧^**OH, consistent with a reduction in the oxidative flux in the system.

### 3.4 Copper-Mediated Oxidation of Trolox^® ^is Inhibited by AAOS

**Figure 5:**
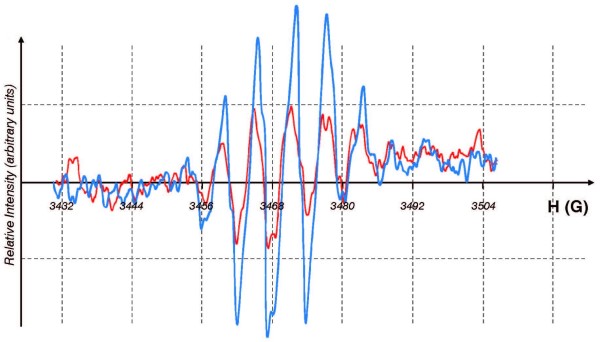
**The EPR spectrum of Trolox^® ^phenoxyl radical**. EPR spectrum of the Trolox^® ^phenoxyl radical formed upon addition of copper to a system of [Trolox^®^] = 2.8 mM and [H_2_O_2_] = 1.7 mM in carbonate buffer, pH 7.2. (blue) copper added as CuSO_4 _(98 μM); (red) copper-AAOS. The isotropic EPR spectrum of the Trolox^® ^phenoxyl radical is described by hyperfine splittings: a(CH_3_) = 5.2 G, and a(CH_3_) = 3.9 G, as reported in [26,33], with other hyperfine splittings not resolvable under our experimental conditions.

**Figure 6:**
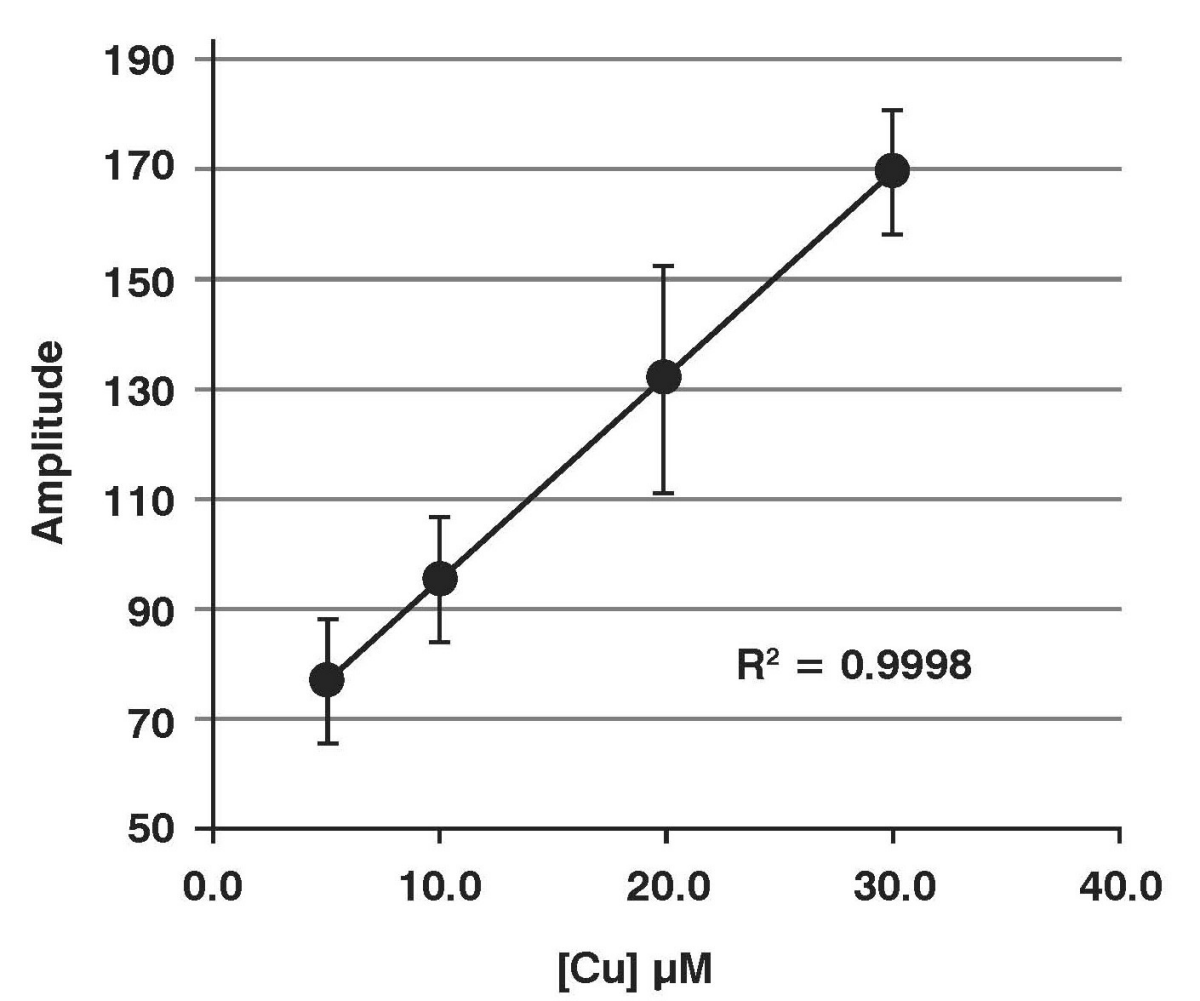
**Trolox^® ^radical formation is directly proportional to [Cu^2+^]**. The linear dose-response curve for addition of varying amounts of CuSO_4 _to this system. (n = 3, standard deviations are shown.)

Trolox^® ^is an analogue of vitamin E, a lipid soluble antioxidant; the phytyl tail of α-tocopherol has been replaced by a carboxyl group making Trolox^® ^water-soluble [25]. It is an excellent tool to probe for antioxidant-capacity and free radical flux [26]. The one-electron oxidation of Trolox^® ^results in the formation of a phenoxyl radical that is readily detected by EPR [27]. Using a Fenton system to initiate oxidations, we observed the formation of the Trolox^® ^free radical upon the introduction of copper, **Figure **5. The concentration of the Trolox^® ^free radical was directly proportional to the amount of Cu^2+ ^introduced into the system, **Figure **6. Thus, this system appears to be appropriate for determining the effectiveness of metals in initiating oxidation processes that will consume antioxidants.

When copper with different coordination environments or matrices was introduced into this system, the intensity of the EPR spectrum of the Trolox^® ^radical varied with the environment. Copper sulfate produced a robust EPR signal of the Trolox^® ^free radical; when gluconate was available to coordinate the copper, the EPR signal was reduced by about 15%; however, when copper was introduced in the AAOS matrix, the EPR signal intensity was reduced by approximately 50%, compared to CuSO_4_. This is consistent with the observations with ascorbate indicating that AAOS reduces the oxidative flux in the system.

### 3.5 The Rate of Oxidation of DCFH_2 _by Copper is Slowed by AAOS

**Figure 7:**
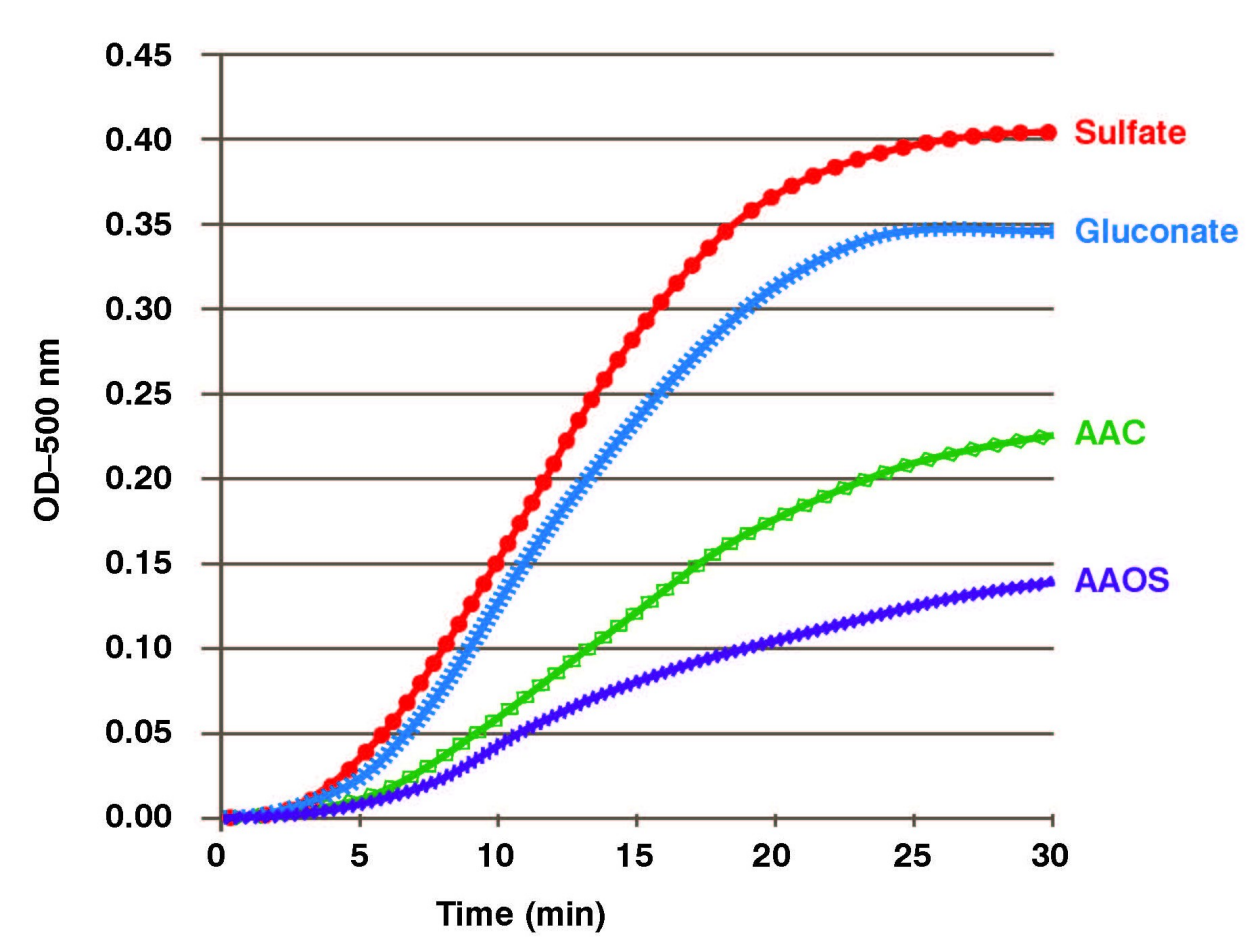
**The rate of oxidation of 2'7'-dichlorodihydrofluorescein by copper is slowed by AAOS**. DCFH_2 _(200 μL of 90 μM in 20 mM carbonate buffer, pH 7.0) and 80 μL of mineral solution (100 μM in 20 mM carbonate, pH 7.0) were mixed in a standard UV-Vis cuvette; total volume 300 μL. The reaction was initiated by addition of 20 μL of 0.3% H_2_O_2 _yielding a final concentration of 88 mM. The formation of DCF was followed by its absorbance at 500 nm.

Another sensitive marker of oxidative flux is 2'7'-dichlorodihydrofluorescein [28]. Its oxidation results in the formation of the two-electron oxidation product, 2'7'-dichlorofluorescein (DCF); DCF can be observed by it absorbance or its fluorescence. Thus, we designed experiments that would follow the kinetics of the oxidation of DCFH_2 _in systems with an oxidative flux. Upon introduction of CuSO_4 _to this system there ensued a rapid oxidation of DCFH_2 _to DCF, **Figure **7. Introduction of copper as a gluconate complex slowed this oxidation marginally. However, AAOS decreased the initial rate of oxidation to just 25% of that observed for CuSO_4_. Thus, AAOS slowed the oxidation of a standard, complementary marker of oxidative flux.

## 3.6 Conclusions

Multivitamin/multimineral complexes are the most common dietary supplements. Besides quality ingredients and the amount of each ingredient in a product, bioavailability is a major concern. Unlike minerals in natural foods that are incorporated in bioorganic structures, minerals in dietary supplements are usually in an inorganic form: sulfates, chlorides, oxides *etc*. The ability of redox active metals to catalyze oxidations is dependent on the coordination environment of the metal. Here we have demonstrated that amino acid complexes of copper (glycinate or aspartate) hinder the catalytic ability of copper or iron compared to the sulfate form of these metals. However, including oligofructose in the matrix brought about an even greater decrease in ability of iron and copper to oxidize substances. Here we have clearly demonstrated that:

• AAOS slows the iron catalyzed oxidation of ascorbate;

• AAOS slows the copper catalyzed oxidation of ascorbate;

• AAOS slows the copper catalyzed oxidation of Trolox^®^, a vitamin E analogue;

• AAOS inhibits the formation of DMPO/**^‧^**OH generated by the Fenton reaction;

• AAOS slows the copper catalyzed oxidation of DCFH_2_, a widely used marker of oxidative flux.

Inulin appears to have many positive health effects [13,29-33]. Our results suggest another positive effect of oligosaccharide in that AAOS offers significant advantages when included in the matrix for the formulation of dietary supplements.

Although not directly addressed in this research, it is reasonable to suggest that how multivitamin/multimineral supplements are formulated can influence the uptake of both the minerals and the vitamins. Here we monitored the oxidations initiated by the redox-active minerals; we not only observed the oxidation of both ascorbate (vitamin C) and Trolox^® ^(a vitamin E analogue) by these minerals, but also surrogate indicators of oxidizing environments, DMPO and DCFH_2_. It can be hypothesized that the oxidations initiated by redox active forms of mineral supplements could also present an oxidative challenge to tissue upon ingestion. Thus, as always there are risks and benefits that need to be understood. Improved formulation of multivitamin/multimineral supplements could both decrease risks and increase benefits.

## 3.7 Abbreviations

AAC: amino acid chelate; such as glycinate; AAOS: amino acid oligosaccharide; DCF: 2',7'-dichlorodifluorescein; DCFH_2_: 2',7'-dichlorodihydrofluorescein; DMPO: 5,5-dimethylpyrroline-1-oxide; EPR: electron paramagnetic resonance.

## 3.8 Competing interests

The authors declare that they have no competing interests.

Melaleuca, Inc. (Idaho Falls, Idaho, United States) provided financial support for this study. AR and JI are employees of Melaleuca, Inc. AK and GB have provided consulting services to Melaleuca, Inc.

## 3.9 Authors contributions

AR, AK, JI, and GB contributed to experimental design. AR and AK ran the experiments. GB, AR and AK contributed to the analysis of the data. AR and GB were principally responsible for writing the paper with assistance from JSI and AMK. All authors read and approved the final manuscript.

## References

[B1] Economic Characterization of the Dietary Supplement Industry U. S. Food and Drug Administration. Center for Food Safety and Applied Nutrition, Section 5 1999

[B2] Buettner GR Ascorbate autoxidation in the presence of iron and copper chelates Free Radic.Res.Commun 1986 1 349 353 10.3109/10715768609051638 2851502

[B3] Buettner GR In the absence of catalytic metals ascorbate does not autoxidize at pH 7: ascorbate as a test for catalytic metals J.Biochem.Biophys.Methods 1988 16 27 40 10.1016/0165-022X(88)90100-5 3135299

[B4] Gallo-Torres HE Machlin LJ Part 5B/Transport and metabolism Vitamin E: A Comprehensive Treatise 1980 New York: Marcel Dekker, Inc 193 267

[B5] Wagner BA Buettner GR Burns CP Free radical-mediated lipid peroxidation in cells: oxidizability is a function of cell lipid bis-allylic hydrogen content Biochemistry 1994 33 4449 4453 10.1021/bi00181a003 8161499

[B6] Khan MM Martell AE Metal ion and metal chelate catalyzed oxidation of ascorbic acid by molecular oxygen. II. Cupric and ferric chelate catalyzed oxidation J.Am.Chem.Soc 1967 89 7104 7111 10.1021/ja01002a046 6064355

[B7] Udenfriend S Clark CT Axelrod J Brodie BB Ascorbic acid in aromatic hydroxylation. I. A model system for aromatic hydroxylation J.Biol.Chem 1954 208 731 739 13174582

[B8] Wagner BA Buettner GR Burns CP Free radical-mediated lipid peroxidation in cells: oxidizability is a function of cell lipid bis-allylic hydrogen content Biochemistry 1994 33 4449 4453 10.1021/bi00181a003 8161499

[B9] Maskos Z Koppenol WH Oxyradicals and multivitamin tablets Free Radic.Biol.Med 1991 11 609 610 10.1016/0891-5849(91)90142-P 1663903

[B10] Fisher AE Naughton DP Iron supplements: the quick fix with long-term consequences Nutr.J 2004 3 2 10.1186/1475-2891-3-2 14728718 PMC340385

[B11] Abrams SA Hawthorne KM Aliu O Hicks PD Chen Z Griffin IJ An inulin-type fructan enhances calcium absorption primarily via an effect on colonic absorption in humans J.Nutr 2007 137 2208 2212 17884999 10.1093/jn/137.10.2208

[B12] Bosscher D Van Caillie-Bertrand M Vanb Cauwenbergh R Deelstra H Availabilities of calcium, iron, and zinc from dairy infant formulas is affected by soluble dietary fibers and modified starch fractions Nutrition 2003 19 641 645 10.1016/S0899-9007(03)00063-7 12831951

[B13] Coudray C Rambeau M Feillet-Coudray C Dietary inulin intake and age can significantly affect intestinal absorption of calcium and magnesium in rats: a stable isotope approach Nutr.J 2005 4 29 10.1186/1475-2891-4-29 16253138 PMC1283151

[B14] Devareddy L Khalil DA Korlagunta K Hooshmand S Bellmer DD Arjmandi BH The effects of fructo-oligosaccharides in combination with soy protein on bone in osteopenic ovariectomized rats Menopause 2006 13 692 699 10.1097/01.gme.0000195372.74944.71 16837891

[B15] Nzeusseu A Dienst D Haufroid V Depresseux G Devogelaer JP Manicourt DH Inulin and fructo-oligosaccharides differ in their ability to enhance the density of cancellous and cortical bone in the axial and peripheral skeleton of growing rats Bone 2006 38 394 399 10.1016/j.bone.2005.09.006 16249132

[B16] Roberfroid MB Cumps J Devogelaer JP Dietary chicory inulin increases whole-body bone mineral density in growing male rats J.Nutr 2002 132 3599 3602 12468594 10.1093/jn/132.12.3599

[B17] Crow JP Dichlorodihydrofluorescein and dihydrorhodamine 123 are sensitive indicators of peroxynitrite in vitro: implications for intracellular measurement of reactive nitrogen and oxygen species Nitric.Oxide 1997 1 145 157 10.1006/niox.1996.0113 9701053

[B18] Basosi R Pogni R Lunga GD Coordination modes of histidine moiety in copper (II) dipeptide complexes detected by multifrequency ESR Bull.Magn.Res 1992 14 224 228

[B19] Buettner GR In the absence of catalytic metals ascorbate does not autoxidize at pH 7: ascorbate as a test for catalytic metals J.Biochem.Biophys.Methods 1988 16 27 40 10.1016/0165-022X(88)90100-5 3135299

[B20] Khan MM Martell AE Metal ion and metal chelate catalyzed oxidation of ascorbic acid by molecular oxygen. I. Cupric and ferric ion catalyzed oxidation J.Am.Chem.Soc 1967 89 4176 4185 10.1021/ja00992a036 6045609

[B21] Buettner GR Jurkiewicz BA Ascorbate free radical as a marker of oxidative stress: an EPR study Free Radic.Biol.Med 1993 14 49 55 10.1016/0891-5849(93)90508-R 8384150

[B22] Sharma MK Buettner GR Interaction of vitamin C and vitamin E during free radical stress in plasma: an ESR study Free Radic. Biol. Med 1993 14 649 653 10.1016/0891-5849(93)90146-L 8392021

[B23] Walling C Fenton's Reagent Revisited Acc.Chem.Res 1975 8 125 131 10.1021/ar50088a003

[B24] Buettner GR Spin trapping: ESR parameters of spin adducts Free Radic.Biol.Med 1987 3 259 303 10.1016/S0891-5849(87)80033-3 2826304

[B25] Scott JW Cort WM Harley H Parrish DR Saucy G 6-Hydroxychroman-2-carboxylic acids: Novel antioxidants J.Am.Oil Chem.Soc 1974 51 200 203 10.1007/BF02632894

[B26] Re R Pellegrini N Proteggente A Pannala A Yang M Rice-Evans C Antioxidant activity applying an improved ABTS radical cation decolorization assay Free Radic.Biol.Med 1999 26 1231 1237 10.1016/S0891-5849(98)00315-3 10381194

[B27] Gilbert BC Kamp NW Smith JRL Oakes J EPR evidence for one-electron oxidation of phenols by a dimeric manganese(IV/IV) triazacyclononane complex in the presence and absence of hydrogen peroxide J.Chem., Perkin Trans.2 1997 2161 2165 10.1039/a704330i

[B28] Hempel SL Buettner GR O'Malley YQ Wessels DA Flaherty DM Dihydrofluorescein diacetate is superior for detecting intracellular oxidants: comparison with 2',7'-dichlorodihydrofluorescein diacetate, 5(and 6)-carboxy-2',7'-dichlorodihydrofluorescein diacetate, and dihydrorhodamine 123 Free Radic.Biol.Med 1999 27 146 159 10.1016/S0891-5849(99)00061-1 10443931

[B29] Abrams SA Hawthorne KM Aliu O Hicks PD Chen Z Griffin IJ An inulin-type fructan enhances calcium absorption primarily via an effect on colonic absorption in humans J.Nutr 2007 137 2208 2212 17884999 10.1093/jn/137.10.2208

[B30] Bosscher D Van Caillie-Bertrand M Van Cauwenbergh R Deelstra H Availabilities of calcium, iron, and zinc from dairy infant formulas is affected by soluble dietary fibers and modified starch fractions Nutrition 2003 19 641 645 10.1016/S0899-9007(03)00063-7 12831951

[B31] Devareddy L Khalil DA Korlagunta K Hooshmand S Bellmer DD Arjmandi BH The effects of fructo-oligosaccharides in combination with soy protein on bone in osteopenic ovariectomized rats Menopause 2006 13 692 699 10.1097/01.gme.0000195372.74944.71 16837891

[B32] Nzeusseu A Dienst D Haufroid V Depresseux G Devogelaer JP Manicourt DH Inulin and fructo-oligosaccharides differ in their ability to enhance the density of cancellous and cortical bone in the axial and peripheral skeleton of growing rats Bone 2006 38 394 399 10.1016/j.bone.2005.09.006 16249132

[B33] Roberfroid MB Cumps J Devogelaer JP Dietary chicory inulin increases whole-body bone mineral density in growing male rats J.Nutr 2002 132 3599 3602 12468594 10.1093/jn/132.12.3599

